# Addressing Palliative Sedation during Expert Consultation: A Descriptive Analysis of the Practice of Dutch Palliative Care Consultation Teams

**DOI:** 10.1371/journal.pone.0136309

**Published:** 2015-08-24

**Authors:** Patrick Hoek, Ilse Grandjean, Constans A. H. H. V. M. Verhagen, Marlies L. E. A. Jansen-Landheer, Henk J. Schers, Cilia Galesloot, Kris C. P. Vissers, Yvonne Engels, Jeroen G.J. Hasselaar

**Affiliations:** 1 Department of Anesthesiology, Pain and Palliative Medicine, Radboud University Medical Center, Nijmegen, the Netherlands; 2 Comprehensive Cancer Centre the Netherlands, Utrecht, the Netherlands; 3 Department of Primary and Community Care, Radboud University Medical Center, Nijmegen, the Netherlands; D'or Institute of Research and Education, BRAZIL

## Abstract

**Main Objective:**

Since palliative sedation is considered a complex intervention, consultation teams are increasingly established to support general practice. This study aims to offer insight into the frequency and characteristics of expert consultations regarding palliative sedation.

**Methods:**

We performed a retrospective analysis of a longitudinal database. This database contained all patient-related consultations by Dutch Palliative Care Consultation teams, that were requested between 2004 and 2011. We described the frequency and characteristics of these consultations, in particular of the subgroup of consultations in which palliative sedation was addressed (i.e. PSa consultations). We used multivariate regression analysis to explore consultation characteristics associated with a higher likelihood of PSa consultations.

**Main Results and Their Significance:**

Of the 44,443 initial consultations, most were requested by general practitioners (73%) and most concerned patients with cancer (86%). Palliative sedation was addressed in 18.1% of all consultations. Palliative sedation was relatively more often discussed during consultations for patients with a neurologic disease (OR 1.79; *95% CI*: *1*.*51–2*.*12*) or COPD (OR 1.39; *95% CI*: *1*.*15–1*.*69)* than for patients with cancer. We observed a higher likelihood of PSa consultations if the following topics were also addressed during consultation: dyspnoea (OR 1.30; *95% CI*: *1*.*22–1*.*40*), agitation/delirium (OR 1.57; *95% CI*: *1*.*47–1*.*68*), exhaustion (OR 2.89; *95% CI*: *2*.*61–3*.*20*), euthanasia-related questions (OR 2.65; *95% CI*: *2*.*37–2*.*96*) or existential issues (OR 1.55; *95% CI*: *1*.*31–1*.*83*).

**Conclusion:**

In conclusion, PSa consultations accounted for almost one-fifth of all expert consultations and were associated with several case-related characteristics. These characteristics may help clinicians in identifying patients at risk for a more complex disease trajectory at the end of life.

## Introduction

Palliative sedation (PS) is commonly applied in end-of-life care, but its frequency and characteristics vary between care settings and countries.[1–8] Palliative sedation involves the deliberate lowering of consciousness in patients nearing death to relieve the burden of refractory symptoms.[9] Refractory symptoms are “symptoms for which all possible treatment has failed, or it is estimated that no methods are available for palliation within the time frame and the risk-benefit ratio that the patient can tolerate”.[10] Because of the far-reaching consequences of palliative sedation, it is considered a treatment option of last resort.[11]

To guide medical practice concerning palliative sedation, several guidelines and frameworks have been published. Expert consultation is one of the topics addressed in these guidelines. For example, the Dutch national guideline on palliative sedation advises consultation with an “appropriate expert” when the attending physician lacks sufficient expertise and experience in the field of palliative sedation.[12] Expert consultation in the context of palliative sedation is also advocated by other frameworks and guidelines on palliative sedation, and some recommend mandatory consultation.[13–19]

Previous research conducted in the Netherlands shows that guideline recommendations on palliative sedation were increasingly applied in daily practice by Dutch physicians.[20] However, in the Netherlands, as well as in other countries, shortcomings were reported regarding the intention to use palliative sedation, the adequate usage of medication and communication with patients, informal caregivers and mutually between formal caregivers.[19–23] Furthermore, Swart et al. demonstrated that 41% of physicians believed that administering palliative sedation had a life-shortening effect and that 14% had experienced pressure to commence sedation, mainly from patients and their relatives.[24] Finally, it has been argued that individual general practitioners typically have little experience with palliative sedation in their caseload.[12,25] Altogether, these findings suggest that there is a substantial need for expert support concerning palliative sedation.

However, if expert consultation services are available, they seem to be used by health care professionals in only a minority of palliative sedation cases.[26–29] Koper et al. investigated considerations of Dutch physicians regarding expert consultations about palliative sedation. Main reasons for physicians to consult specialist palliative care services for palliative sedation are a lack of expertise and that consultation is considered supportive. Reasons not to use these consultation services were as follows: physicians having sufficient expertise themselves; consideration of palliative sedation as a normal medical procedure; time pressure; fear of disagreement with the consultant; and considering consultation as having little added value.[30] An alternative approach to gain additional insight in consultation practices concerning palliative sedation is to study, nationwide, the documentation of requested consultations at expert consultation services.

Therefore, the aims of this study are to investigate the following over a period of 8 years: 1) the frequency and characteristics of palliative care consultations given by Dutch Palliative Care Consultation (PCC) teams, 2) the frequency and characteristics of consultations in which PS was addressed, and 3) the consultation characteristics associated with a higher likelihood of PS being addressed during consultation. These characteristic might help clinicians in identifying patients at risk for a more complex disease trajectory at the end of life, for whom timely consultation with an expert might be appropriate.

## Materials and Methods

### Design

We performed a retrospective analysis of a national database consisting of the registration of all consultations by Dutch PCC teams that were requested between 2004 and 2011.

### Setting

In 1998, the Dutch government installed a national program for the development and improvement of palliative care. As a result of this program, regional PCC teams were established to ensure the availability of specific advice concerning palliative care for all professional caregivers. In 2011, 23 PCC teams were available for expert consultation, covering the whole country. PCC teams generally consist of general practitioners, medical specialists and specialized nurses who are recruited from regional health centers and hospitals. PCC team members are practicing healthcare professionals who have a special interest in palliative care and are trained in such. Some PCC teams offer a 24/7-consultation service, while others are available for consultation only during weekdays and to a limited extent during out-of-office hours. Most consultations were requested and answered by phone (approximately 90% in 2011).[31] Often, advice given by consultants is previously discussed with one or more fellow consultants.[31,32] Recommendations are mainly communicated by phone and, as much as possible, confirmed in writing. Only in a small minority of cases bedside consultations occur.[31] For each individual consultation, consultants themselves registered their specific clinical findings, diagnosis, treatment advice and other appointments in a national, standardized, web-based, consultation database (*PRADO*, *IKNL*, *Ecommany*, *the Netherlands*).

### Participants

In PRADO, a number of characteristics were systematically recorded for every consultation, mainly using tick boxes: date of consultation, region of consultation, patient demographics (date of birth, age, gender), diagnosis, estimated prognosis, listed symptoms and problems, place of residence and the specialty or training of the professional caregiver requesting the consultation. With regard to the listed problems, within PRADO the following sentence is displayed: “the consultation concerns the following problems…”. Subsequently, the consultant lists one or more problems that have been addressed during consultation, using tick boxes. “Palliative sedation”, is one of the options available in this list.

We grouped consultations based on whether or not “palliative sedation” was selected by the consultant. As a result, two groups were defined: 1) consultations in which palliative sedation was addressed by the consulting caregiver and/or the consultant and registered as such in PRADO (PS addressed, abbreviated as PSa consultations), 2) other consultations (PS not addressed, abbreviated as PSna consultations). To prevent duplication of cases, we included only the first consultation for every patient.

### Statistical analysis and main outcome measures

Data were exported from the PRADO system into a Comma-Separated Value (CSV) file. Data were anonymized and de-identified and were subsequently imported and analyzed in Stata, Version 12.0 *(StataCorp*. *2011*. *Stata Statistical Software*: *Release 12*. *StataCorp LP*, *College Station*, *Texas*, *United States*).

The number of annual consultations for PCC teams was described and displayed as a percentage of the annual mortality rates as registered by Statistics Netherlands (CBS). Proportions and 95% confidence intervals (95% CIs) were used to describe differences in characteristics between PSa consultations and PSna consultations. Differences in characteristics were analyzed for statistical significance using uni- and multivariate regression analysis. A p-level < 0.05 was considered statistically significant.

### Ethical considerations

The Committee on Research Involving Human Subjects (CMO) Region Arnhem-Nijmegen stated that no further ethical assessment was required for this research. (Reg. 2013/423). Informed consent was not obtained, since we only used data from an existing, clinical database, that was anonymized and de-identified prior to analysis.

## Results

### General description of all palliative care consultations

During the study period, 44,443 first consultations were registered and included for analysis. The vast majority of these consultations were requested by general practitioners (GPs) (72.7%) and concerned patients residing at home (74.7%). Most patients enlisted for consultation were diagnosed with cancer (86.1%). The most frequently listed symptoms were pain (45.0%), agitation/delirium (15.4%), dyspnoea (15.4%), and nausea/vomiting (15.0%). Other topics frequently addressed during consultation related to pharmacological questions (51.2%), moral support for the professional caregivers themselves (33.7%) and issues about the organization of care (23.7%). More than half of the consultations (56.8%) concerned patients with an estimated prognosis of less than 4 weeks. The vast majority of consultations were requested during office hours (85.0%) (Table 1). The annual consultation rate, in relation to the annual mortality number, rose during the first years of the study, from 3.6% (*95% CI*: *3*.*5%- 3*.*7%)* in 2004 to 4.8% *(95% CI*: *4*.*7%-4*.*9%)* in 2006. After 2006, annual consultation rates declined until 2008 and remained rather stable at approximately 4% afterwards (Table 2).

**Table 1:** Characteristics of all consultations by Dutch PCC teams (2004–2011).

Variable	All consultations		PSa consultations		Proportion of PSa consultations*	
	n	%	n	%	%	(95% CI)
**Year of consultation**						
*2004*	4,851	10.9	509	6.3	10.5	(9.6–11.4)
*2005*	5,508	12.4	735	9.1	13.3	(12.4–14.2)
*2006*	6,465	14.6	1,178	14.6	18.2	(17.3–19.2)
*2007*	6,044	13.6	1,145	14.2	18.9	(18.0–19.9)
*2008*	5,283	11.9	1,127	14.0	21.3	(20.2–22.4)
*2009*	5,532	12.5	1,111	13.8	20.1	(19.0–21.1)
*2010*	5,638	12.7	1,125	14.0	20.0	(18.9–21.0)
*2011*	5,122	11.5	1,108	13.8	21.6	(20.5–22.8)
**Consultation region**						
*North*	5,728	12.9	924	11.5	16.1	(15.2–17.1)
*East*	11,536	26.0	1,577	19.6	13.7	(13.0–14.3)
*South*	8,591	19.3	1,460	18.2	17.0	(16.2–17.8)
*West*	10,760	24.2	2,011	25.0	18.7	(18.0–19.4)
*Middle*	7,828	17.6	2,066	25.7	26.4	(25.4–27.4)
**Consulting Professional Caregiver**						
*General practitioner*	32,319	72.7	7,023	87.4	21.7	(21.3–22.2)
*Hospital specialist*	2,666	6.0	196	2.4	7.4	(6.4–8.3)
*Nurse*	5,183	11.7	331	4.1	6.4	(5.7–7.1)
*Elderly care physician*	1,674	3.8	244	3.0	14.6	(12.9–16.3)
*Other*	2,586	5.8	242	3.0	9.4	(8.2–10.5)
*Unknown*	15	0.03	2	0.02	13.3	(-3.9–30.5)
**Place of residence**						
*Home*	33,190	74.7	6,435	80.1	19.4	(19.0–19.8)
*Hospice*	2,874	6.5	516	6.4	18.0	(16.6–19.4)
*Nursing/Residential Home*	3,328	7.5	683	8.5	20.5	(19.2–21.9)
*Hospital*	4,008	9.0	271	3.4	6.8	(6.0–7.5)
*Other*	928	2.1	121	1.5	13.0	(10.9–15.2)
*Unknown*	115	0.3	12	0.2	10.4	(4.8–16.0)
**Gender**						
*Male*	22,593	50.8	4,218	52.5	18.7	(18.2–19.2)
*Female*	21,804	49.1	3,813	47.4	17.5	(17.0–18.0)
*Unknown*	46	0.1	7	0.1	15.2	(4.8–25.6)
**Age (years)**						
*0–39*	1,473	3.3	258	3.2	17.5	(15.6–19.5)
*40–64*	16,189	36.4	2,749	34.2	17.0	(16.4–17.6)
*65–79*	16,655	37.5	2,947	36.7	17.7	(17.1–18.3)
*≥80*	9,435	21.2	1,969	24.5	20.9	(20.0–21.7)
*Unknown*	691	1.6	115	1.4	16.6	(13.9–19.4)
**Diagnosis**						
*Cancer*	38,248	86.1	6,597	82.1	17.2	(16.9–17.6)
*Heart failure*	1,135	2.6	284	3.5	25.0	(22.5–27.5)
*Neurologic disease*	1,065	2.4	244	3.0	22.9	(20.4–25.4)
*Chronic obstructive pulmonary disease (COPD)*	694	1.6	183	2.3	26.4	(23.1–29.6)
*Other*	2,477	5.6	590	7.3	23.8	(22.1–25.5)
*Unknown*	824	1.9	140	1.7	17.0	(14.4–19.6)
**Symptoms** ^b^						
*Anorexia*	1,842	4.1	273	3.4	14.8	(13.2–16.4)
*Dyspnoea*	6,858	15.4	1,728	21.5	25.2	(24.2–26.2)
*Agitation/delirium*	6,857	15.4	1,937	24.1	28.2	(27.2–29.3)
*Decubitus/ulcus*	1,024	2.3	121	1.5	11.8	(9.8–13.8)
*Depressed mood*	3,268	7.4	370	4.6	11.3	(10.2–12.4)
*Nausea/vomiting*	6,680	15.0	899	11.2	13.5	(12.6–14.3)
*Mouth problems*	1,149	2.6	156	1.9	13.6	(11.6–15.6)
*Constipation/diarrhea*	2,168	4.9	206	2.6	9.5	(8.3–10.7)
*Pain*	19,977	45.0	2,924	36.4	14.6	(14.1–15.1)
*Exhaustion*	2,981	6.7	857	10.7	28.7	(27.1–30.4)
*Other*	8,412	18.9	1,463	18.2	17.4	(16.6–18.2)
**Issues** ^b^						
*Daily functioning*	5,005	11.3	534	6.6	10.7	(9.8–11.5)
*Euthanasia-related*	1,981	4.4	682	8.5	34.4	(32.3–36.5)
*Pharmacological*	22,739	51.2	3,514	43.7	15.5	(15.0–15.9)
*Informal care*	3,501	7.9	429	5.3	12.3	(11.2–13.3)
*Moral support professional caregiver*	14,992	33.7	2,386	29.7	15.9	(15.3–16.5)
*Organization of care*	10,529	23.7	1,409	17.5	13.4	(12.7–14.0)
*Social*	2,593	5.8	249	3.1	9.6	(8.5–10.7)
*Existential*	1,175	2.6	254	3.2	21.6	(19.3–24.0)
*Other*	2,428	5.5	204	2.5	8.4	(7.3–9.5)
**Estimated Prognosis**						
*Less than 4 weeks*	25,231	56.8	6,898	85.8	27.3	(26.8–27.9)
*Between 4 weeks and 3 months*	7,600	17.1	333	4.1	4.4	(3.9–4.8)
*More than 3 months*	3,394	7.6	82	1.0	2.4	(1.9–2.9)
*Unknown*	8,218	18.5	725	9.0	8.8	(8.2–9.4)
**Time of consultation**						
*Out-of-office hours*	6,377	14.4	1,497	18.6	23.5	(22.4–24.5)
*Office hours*	37,763	85.0	6,509	80.1	17.2	(16.9–17.6)
*Unknown*	303	0.7	32	0.4	10.6	(7.1–14.0)

**PCC teams**: Palliative Care Consultation Team; **PS**: palliative sedation; **PSa consultation**: palliative sedation addressed during consultation; **95% CI**: 95% confidence interval.

^a^ Proportion = (PSa consultations / total consultations)*100%, per single variable.

^b^ Total of variables for “Symptoms” and “Issues” exceeds 100% due to more than one symptom / issue registered per consultation.

**Table 2:** Number of all consultations by Dutch PCC teams over the period 2004–2011.

	AMN	All consultations			PSa consultations		
	n	n	% of AMN	(95% CI)	n	% of AMN	(95% CI)
**Year of consultation**							
*2004*	136,553	4,851	3.6	(3.5–3.7)	509	0.4	(0.3–0.4)
*2005*	136,402	5,508	4.0	(3.9–4.1)	735	0.5	(0.5–0.6)
*2006*	135,372	6,465	4.8	(4.7–4.9)	1,178	0.9	(0.8–0.9)
*2007*	133,022	6,044	4.5	(4.4–4.7)	1,145	0.9	(0.8–0.9)
*2008*	135,136	5,283	3.9	(3.8–4.0)	1,127	0.8	(0.8–0.9)
*2009*	134,235	5,532	4.1	(4.0–4.2)	1,111	0.8	(0.8–0.9)
*2010*	136,058	5,638	4.1	(4.0–4.3)	1,125	0.8	(0.8–0.9)
*2011*	135,741	5,122	3.8	(3.7–3.9)	1,108	0.8	(0.8–0.9)

**PCC teams**: Palliative Care Consultation Teams; **AMN**: Annual Mortality Number; **PS**: palliative sedation; **PSa consultation**: palliative sedation addressed during consultation; **95% CI**: 95% confidence interval.

### PSa consultations

Palliative sedation was addressed in 8,038 (18.1%) of the consultations. PSa consultations were most often requested by GPs (87.4% of all PSa consultations, 7,023 PSa consultations) and most often concerned patients diagnosed with cancer (82.1%, 6,597 PSa consultations). Since 86.1% (38,248 consultations) of all consultations concerned patients diagnosed with cancer, the proportion of PSa consultations for this group of patients was relatively low (17.2%; *95% CI*: *16*.*9%-17*.*6%*). Relatively high proportions of PSa consultations were found for patients diagnosed with heart failure (25.0%; *95% CI*: *22*.*5%-27*.*5%*), neurologic diseases (22.9%; *95% CI*: *20*.*4%-25*.*4%*), chronic obstructive pulmonary disease (COPD) (26.4%; *95% CI*: *23*.*1%-29*.*6%*), and other diagnoses (23.8%; *95% CI*: *22*.*1%-25*.*5%*). Furthermore, palliative sedation was relatively often addressed in consultations in which the following symptoms or issues were also raised: exhaustion (proportion of PSa consultations: 28.7%; *95% CI*: *27*.*1%-30*.*4%*), agitation/delirium (28.2%; *95% CI*: *27*.*2%-29*.*3%*), dyspnoea (25.2%; *95% CI*: *24*.*2%-26*.*2%*), euthanasia-related (34.4%; *95% CI*: *32*.*3%-36*.*5%*), and existential issues (21.6%; *95% CI*: *19*.*3%-24*.*0%*). Also, a relatively high proportion of PSa consultations was found for patients with an estimated prognosis of less than 4 weeks (27.3%; *95% CI*: *26*.*8%-27*.*9%*). Finally, palliative sedation was addressed relatively often in consultations outside of office hours (23.5%; *95% CI*: *22*.*4%-24*.*5%*) (Table 1). Over the years, palliative sedation was increasingly addressed during PCC consultations, from 10.5% of cases in 2004 (*95% CI*: *9*.*6%-11*.*4%)* to 21.6% of cases in 2011 (*95% CI*: *20*.*5%- 22*.*8%)*. PSa consultation rates as a percentage of annual mortality numbers varied between 0.4% in 2004 (*95% CI*: *0*.*3%-0*.*4%)* to 0.9% in 2006 and 2007 (*95% CI*: *0*.*8%-0*.*9%* for both years) (Table 2).

### Multivariate regression analysis—characteristics associated with PSa consultations

The following characteristics were associated with a higher likelihood of palliative sedation being addressed during palliative care consultations: 1) patients being diagnosed with a neurologic disease (OR 1.79, *95% CI*: *1*.*51–2*.*12*) or COPD (OR1.39, *95% CI*: *1*.*15–1*.*69*) compared to cancer (*reference variable*) and 2) the presence of dyspnoea (OR 1.30, *95% CI*: *1*.*22–1*.*40*), agitation/delirium (OR 1.57; *95% CI*: *1*.*47–1*.*68*), exhaustion (OR 2.89; *95% CI*: *2*.*61–3*.*20*), euthanasia-related issues (OR 2.65; *95% CI*: *2*.*37–2*.*96*) or existential issues (OR 1.55; *95% CI*: *1*.*31–1*.*83*). The following characteristics were associated with a lower likelihood of palliative sedation being addressed during consultations: 1) patients having a prognosis of more than 4 weeks ((OR 0.15 (*95% CI*: *0*.*13–0*.*16)* for an estimated prognosis of “between 4 weeks and 3 months” and 0.08 *(95% CI*: *0*.*06–0*.*10)* for “more than 3 months,”, respectively), 2) professional caregivers other than the GP being the caregivers requesting consultation (OR varying from 0.36 for nurses (*95% CI*: *0*.*32–0*.*41*) to 0.61 for elderly care physicians (*95% CI*: *0*.*51–0*.*72)*) and 3) the timing of the consultation characterized as “during office hours” (OR 0.88; *95% CI*: *0*.*82–0*.*95*) compared to “out-of-office hours” *(reference variable)* (Table 3).

**Table 3:** Univariate and multivariate analysis of all consultations by Dutch PCC teams (2004–2011)–Characteristics associated with PSa consultations.

	Univariate analysis	Multivariate analysis
	*Odds ratio (95% CI)*	*Odds ratio (95% CI)*
**Year of consultation**		
*2004*	1 (reference)	1 (reference)
*2005*	1.31 (1.16–1.48)	1.41 (1.24–1.61)
*2006*	1.90 (1.70–2.12)	1.69 (1.49–1.91)
*2007*	1.99 (1.78–2.23)	1.66 (1.47–1.88)
*2008*	2.31 (2.07–2.59)	2.26 (1.99–2.57)
*2009*	2.14 (1.91–2.40)	2.11 (1.86–2.40)
*2010*	2.13 (1.90–2.38)	1.98 (1.74–2.24)
*2011*	2.35 (2.10–2.64)	2.17 (1.91–2.47)
**Consultation region**		
*North*	1 (reference)	1 (reference)
*East*	0.82 (0.75 0.90)	0.90 (0.81–0.99)
*South*	1.06 (0.97–1.16)	1.28 (1.15–1.41)
*West*	1.20 (1.09–1.30)	1.19 (1.08–1.31)
*Middle*	1.86 (1.71–2.03)	1.42 (1.29–1.57)
**Consulting professional caregiver**		
*General practitioner*	1 (reference)	1 (reference)
*Hospital specialist*	0.29 (0.25–0.33)	0.52 (0.42–0.64)
*Nurse*	0.25 (0.22–0.28)	0.36 (0.32–0.41)
*Elderly care physician*	0.61 (0.54–0.71)	0.61 (0.51–0.72)
*Other*	0.37 (0.32–0.43)	0.52 (0.45–0.61)
*Unknown*	0.55 (0.13–2.46)	0.78 (0.17–3.70)
**Place of residence**		
*Home*	1 (reference)	1 (reference)
*Hospice*	0.91 (0.82–1.00)	1.09 (0.97–1.22)
*Nursing/Residential home*	1.07 (0.98–1.17)	1.02 (0.91–1.14)
*Hospital*	0.30 (0.27–0.34)	0.70 (0.59–0.84)
*Other*	0.62 (0.51–0.76)	0.85 (0.69–1.05)
*Unknown*	0.48 (0.27–0.88)	0.83 (0.43–1.58)
**Gender**		
*Male*	1 (reference)	1 (reference)
*Female*	0.92 (0.88–0.97)	0.98 (0.93–1.03)
*Unknown*	0.78 (0.35–1.75)	1.11 (0.44–2.78)
**Age (years)**		
*0–39*	1 (reference)	1 (reference)
*40–64*	0.96 (0.84–1.11)	0.93 (0.80–1.09)
*65–79*	1.01 (0.88–1.16)	0.93 (0.79–1.08)
*≥ 80*	1.24 (1.07–1.43)	0.93 (0.80–1.10)
*Unknown*	0.94 (0.74–1.20)	1.09 (0.83–1.43)
**Diagnosis**		
*Cancer*	1 (reference)	1 (reference)
*Heart failure*	1.60 (1.40–1.84)	1.15 (0.98–1.34)
*Neurologic disease*	1.43 (1.23–1.65)	1.79 (1.51–2.12)
*Chronic obstructive pulmonary disease (COPD)*	1.72 (1.45–2.04)	1.39 (1.15–1.69)
*Other*	1.50 (1.36–1.65)	1.53 (1.37–1.72)
*Unknown*	0.98 (0.82–1.18)	1.06 (0.87–1.31)
**Symptoms (present)**		
*Anorexia*	0.78 (0.68–0.89)	1.13 (0.97–1.32)
*Dyspnoea*	1.67 (1.57–1.77)	1.30 (1.22–1.40)
*Agitation/delirium*	2.03 (1.91–2.16)	1.57 (1.47–1.68)
*Decubitus/ulcus*	0.60 (0.50–0.73)	0.87 (0.70–1.08)
*Depressed mood*	0.56 (0.50–0.62)	0.96 (0.84–1.09)
*Nausea/vomiting*	0.67 (0.62–0.72)	0.72 (0.67–0.79)
*Mouth problems*	0.71 (0.60–0.84)	0.92 (0.76–1.11)
*Constipation/diarrhea*	0.46 (0.40–0.53)	0.66 (0.56–0.77)
*Pain*	0.65 (0.62–0.68)	0.74 (0.70–0.78)
*Exhaustion*	1.93 (1.77–2.09)	2.89 (2.61–3.20)
*Other*	0.94 (0.89–1.00)	1.00 (0.93–1.08)
**Issues (present)**		
*Daily functioning*	0.51 (0.46–0.56)	0.76 (0.69–0.85)
*Euthanasia-related*	2.51 (2.28–2.76)	2.65 (2.37–2.96)
*Pharmacological*	0.69 (0.66–0.73)	0.54 (0.51–0.58)
*Informal care*	0.61 (0.55–0.68)	1.09 (0.97–1.24)
*Moral support professional caregiver*	0.80 (0.76–0.84)	0.84 (0.79–0.90)
*Organization of care*	0.64 (0.60–0.68)	0.84 (0.78–0.90)
*Social*	0.46 (0.41–0.53)	0.87 (0.74–1.02)
*Existential*	1.26 (1.09–1.45)	1.55 (1.31–1.83)
*Other*	0.40 (0.35–0.46)	0.46 (0.39–0.53)
**Prognosis**		
*Less than 4 weeks*	1 (reference)	1 (reference)
*Between 4 weeks and 3 months*	0.12 (0.11–0.14)	0.15 (0.13–0.16)
*More than 3 months*	0.07 (0.05–0.08)	0.08 (0.06–0.10)
*Unknown*	0.26 (0.24–0.28)	0.26 (0.24–0.28)
**Time of consultation**		
*Out-of-office hours*	1 (reference)	1 (reference)
*Office Hours*	0.68 (0.64–0.72)	0.88 (0.82–0.95)
*Unknown*	0.38 (0.27–0.56)	1.02 (0.68–1.53)

**PCC teams**: Palliative Care Consultation Teams; **PS**: palliative sedation; **PSa consultation**: palliative sedation addressed during consultation; **95% CI**: 95% confidence interval.

## Discussion

### Main results

Palliative sedation was addressed in approximately one out of five palliative care consultations in the Netherlands during the period 2004–2011. The likelihood of palliative sedation being addressed during consultations was higher if the consultations were requested by GPs and during “out of office” hours. Additionally, palliative sedation was relatively more often addressed for patients with a non-cancer diagnosis, for patients with an estimated prognosis of less than 4 weeks, and for patients with dyspnoea, agitation/delirium, exhaustion, euthanasia-related or existential issues.

### Strengths and weaknesses

A main strength of this study is the use of a national, standardized database containing all consultations for Dutch PCC teams in the Netherlands that were provided and registered in the period 2004 to 2011. By using this database, we were able to give a detailed overview of the practice of palliative care consultations during this period. The number of missing values is low (<2% of all cases per variable) for most variables. However, Schrijnemakers et al. demonstrated that during telephone consultation a substantial share of the present problems may not be discussed and subsequently not be registered.[32]

Furthermore, to prevent duplication of patients with multiple consultations in our study sample, we decided to include only the first consultation per patient. Consequently, follow-up consultations in which palliative sedation may have been addressed were not included. Nevertheless, our final sample included 89.3% of all PSa consultations (8,038 out of 9,005 PSa consultations) by Dutch PCC teams, which warrants the representativeness of our results. Another consequence of the study methods used is that we were not able to study which percentage of PSa consultations eventually led to the actual application of palliative sedation. Furthermore, palliative sedation was defined in a general way, therefore no distinction could be made between continuous and intermittent sedation. These factors hamper a comparison of our study to the international literature concerning the practice and incidence of palliative sedation. Also, since palliative sedation could only be registered by checking the tick-box “palliative sedation”, no additional information was available on this topic (e.g. who initiated discussion about palliative sedation?). Therefore PSa consultations may form a heterogeneous group of consultations.

Finally, consultation rates from this study must be interpreted with caution because we have studied only consultations delivered by PCC teams. Consequently, we did not take into account alternative sources for palliative care consultations (e.g., hospital specialists or colleagues specialized in palliative care). Rietjens et al. showed that physicians, particularly clinical specialists, but also 29% of the GPs, discuss sedation with other physicians rather than with a consultation service.[29] These “unofficial” consultations are not taken into account in our study. This may have led to an underestimation of consultation rates.

### Comparison with the literature

We found an increase in PSa consultations over the period from 2004 to 2011. This appears to be in line with previous research describing the incidence of palliative sedation in the Netherlands and Belgium.[3,4,33] The sharpest increase can be observed between 2005 and 2006. This finding may be related to the publication of the guidelines on palliative sedation by the Royal Dutch Medical Association (RDMA) in 2005, most likely leading towards an increased awareness among practitioners.[20] In the Netherlands, the estimated incidence of (continuous) palliative sedation in 2010 was 12.3% of all deaths.[3] Similar or higher percentages were found in Belgium (14.5% in 2007) and the United Kingdom (18.7% in 2007/2008).[4,23,33] In our study, the PSa consultation rate (PSa consultations as a percentage of the annual number of deaths) in 2010 was 0.8%. This suggests one PSa consultation by PCC teams for every 15 cases of palliative sedation in the Netherlands (12.3%/0.8%). This rate is lower than the consultation rate of 22% found by Swart et al.[24] One explanation for this different consultation rate, could be that Swart et al. asked physicians whether they consulted any palliative care team, whereas we only studied consultations requested at PCC teams. Anyhow, our finding demonstrates that the use of PCC teams for palliative sedation seems not a common practice. This rate is probably higher for general practitioners, as PSa consultations in our study were mainly requested by general practitioners (87.4% of all PSa consultations), while, according to the literature, general practitioners only perform almost half of all sedations.[34]

Our study raised three further topics that warrant discussion. First, dyspnoea, agitation/delirium and exhaustion were significantly associated with a higher likelihood of palliative sedation being addressed during consultation compared to other listed symptoms. A systematic review by Maltoni et al. showed that the most common indication for palliative sedation is delirium (54%) followed by dyspnoea (30%).[35] The complex management of these symptoms in end of life care is acknowledged in literature.[36,37] The finding that exhaustion is associated with a higher likelihood of PSa consultations is in line with other research conducted in the Netherlands, stating that exhaustion is a common indication to start palliative sedation.[20,24] However, it is not mentioned as a common indication for palliative sedation in recent international literature.[35] Furthermore, although pain is a commonly mentioned symptom during PSa consultations (2,924 PSa consultations; 36.4%), resonating with the literature,[20,24,25] it is not independently associated with a higher likelihood of PSa consultations. This might be a result of the overall high incidence of pain in all consultations requested at PCC teams (19,977 consultations; 45.0%). As a result of this high denominator, the proportion of PSa consultations is relatively low for pain.

Second, apart from symptoms, the underlying diagnosis appears relevant. Although cancer accounts for the vast majority of palliative care consultations,[38] palliative sedation was discussed relatively more often for patients diagnosed with COPD and neurologic diseases. This might be explained by the fact that patients with these diagnoses experience a relatively unpredictable disease trajectory.[39,40] On the one hand, this may result in an increased risk of a complex terminal trajectory with a higher chance at refractory symptoms and PSa consultation. On the other hand, this may result in uncertainty regarding the estimated prognosis of patients and the suitability of palliative sedation. It is therefore important that clinicians confronted with patients with an end-stage COPD or neurologic disease prepare advanced care planning for palliative care in time, including expert involvement.

Third, consultations in which existential issues were discussed were associated with a higher likelihood of palliative sedation being addressed during consultation. The RDMA guideline on palliative sedation recognizes that existential suffering may be among the refractory symptoms that lead to unbearable suffering and state that expert consultations for these problems are strongly recommended.[12] However, existential suffering as an indication for palliative sedation remains controversial.[41] Previous research has shown that euthanasia, rather than palliative sedation, is related to existential problems relatively often.[42] In this regard, it is also important to notice that addressing “euthanasia-related issues” and palliative sedation were highly interrelated in this study. PCC teams appear to have a role in supporting physicians to explore palliative care options for patient cases in which questions concerning euthanasia, existential issues and palliative sedation intermingle. Clinicians confronted with existential suffering in end-of-life care are therefore encouraged to seek support from expert teams to prevent palliative sedation being applied for controversial indications.

## Conclusion

Expert consultation in the field of palliative sedation is advocated when physicians lack sufficient knowledge or expertise in the field of palliative sedation. However, mandatory consultation for palliative sedation, as argued by some, remains an ongoing debate in the recent literature.[30,43] From our study, the use of expert consultation services for palliative sedation does not appear to be a common practice. This might indicate a *limited* need for expert consultation as a result of physicians being sufficiently skilled in this area. It might also point to an already *covered* need for expert consultation provided by other healthcare professionals beyond regular consultation services. Finally, there might be a *neglected* need for expert consultations due to physicians being unaware of their limited expertise concerning palliative sedation. Further research is needed here.

Consultation rates for palliative sedation appear to be rather low. To verify the accuracy of these consultation rates, future research must reveal the use of expert consultation services other than established PCC teams. In addition, future research should yield more insight into the needs of general practitioners concerning palliative sedation, which could guide current palliative care consultation teams in adapting their services to these needs. This might lead to an increase in consultation rates and eventually improve the practice of palliative sedation.

## Supporting Information

S1 File Additional information about the multivariate regression analysis. (XLSX)
